# Computed tomography for evaluating right ventricle and pulmonary artery in pediatric tetralogy of Fallot: correlation with post-operative pulmonary regurgitation

**DOI:** 10.1038/s41598-018-25868-w

**Published:** 2018-05-14

**Authors:** Yue Gao, Zhi-gang Yang, Ke Shi, Kai-yue Diao, Hua-yan Xu, Ying-kun Guo

**Affiliations:** 1 Department of Radiology, West China Hospital, Sichuan University, 37# Guo Xue Xiang, Chengdu, Sichuan 610041 China; 2 Department of Radiology, West China Second University Hospital, Sichuan University, 20# Section 3 South Renmin Road, Chengdu, Sichuan 610041 China

## Abstract

Pulmonary regurgitation (PR) is the most common complication after tetralogy of Fallot (TOF) surgical repair, and long-term PR might result in cardiovascular events. The aim of this study was to assess the influence of pre-operative right ventricle (RV) and pulmonary artery (PA) parameters assessed by dual-source computed tomography on post-operative PR. A total of 41 TOF patients who underwent trans-valve surgical repair were retrospectively recruited. The RV and PA parameters evaluated by pre-operative DSCT were compared between the PR and non-PR groups. Our result revealed that the PA parameters (McGoon ratio, Nakata index, and LPA diameter) and RV parameters (RV length diameter and RV short diameter) all showed significant differences between the two groups (all p < 0.05). There was a significant correlation between PR and LPA diameter (r = 0.361), McGoon ratio (r = 0.413), and Nakata index (r = 0.482). Receiver operating characteristic analysis also revealed a moderate sensitivity and specificity of LPA (66.33%; 82.60%), McGoon ratio (83.33%, 56.52%), and Nakata index (83.33%; 60.87%) for predicting the occurrence of PR. This study indicated that these pre-operative indices calculated by DSCT are associated with post-operative PR and that these pre-operative PA and RV parameters may serve as novel predictors of the risk of PR.

## Introduction

Tetralogy of Fallot (TOF) is one of the most common cyanotic congenital heart diseases (CHDs), and pulmonary regurgitation (PR) is the most common complication after TOF surgical repair. Long-standing PR may result in chronic right ventricle (RV) volume overload, ventricular dysfunction, arrhythmia, and adverse cardiovascular events^1^. Severe PR after TOF surgical repair requires reoperation. Thus, the ability to accurately predict the occurrence of PR may help early monitor and help improve patient prognosis.

Trans-thoracic echocardiography (TTE) as the traditional and routine clinical investigation for TOF, is good at hemodynamic detection; however, the results of TTE are largely dependent on the operators. Dual-source computed tomography (DSCT) post-processing techniques for the heart and vessels, which enables better anatomical delineation of cardiac structures at a higher spatial resolution and lower radiation dose compared to older generation CT scanners, are more widely used in CHD^2–5^.

In recent years, numerous TOF-related studies have focused on the clinical managements after the TOF surgical repair, such as the close monitoring of ventricle function changes in the long-term postoperative follow-up, discussion about indications, methods, and optimal timing of pulmonary valve replacement (PVR) have persisted^6–10^. Although study has pointed out that trans-valve surgical repair has been linked with post-operative PR, the preoperative anatomic characteristics, which play a major role in deciding the surgical approach and the response of the perivalvular structures to the hemodynamic load have not been thoroughly discussed^11–13^.

In this study, we collected fifty-five patients to assess the influence of pre-operative RV and PA imaging characteristics by DSCT on post-operative PR.

## Results

### Patient characteristics

A total of 55 patients who did not pre-operative PR were enrolled in the study, these included 20 patients in whom post-operative PR was confirmed by TTE and 35 patients who did not have postoperative PR. Of the 55 patients, there were 41 patients had received trans-valve surgery. In the post-operative PR group, 18 patients had received trans-valve surgery (90.0%), and in the non-PR group, 23 patients had received trans-valve surgery.

The demographic and clinical data for patients with or without PR are as given below: age (1.58 ± 1.12 years vs. 1.74 ± 1.45 years; p = 0.665), systolic pressure (95.80 ± 13.45 mmHg vs. 95.58 ± 21.87 mmHg; p = 0.967), and diastolic pressure (53.70 ± 8.55 mmHg vs. 57.76 ± 17.25 mmHg; p = 0.33) (Table 1).

**Table 1:** Baseline characteristics of TOF patients.

	PR (n = 20)	non-PR (n = 35)	P value
Age, y	1.58 ± 1.12	1.74 ± 1.45	0.665
Male gender, n	10(50%)	19(54.3%)	0.803
Body surface area, m^2^	0.46 ± 0.96	0.47 ± 0.11	0.734
Heart rate, bpm	128.75 ± 21.36	127.31 ± 15.70	0.776
Systolic pressure, mmHg	95.80 ± 13.45	95.58 ± 21.87	0.967
Diastolic pressure, mmHg	53.70 ± 8.55	57.76 ± 17.25	0.332
Height, cm	73.48 ± 2.637	73.44 ± 7.40	0.982
Weight, kg	9.41 ± 2.11	10.27 ± 3.18	0.248
Trans-valve surgery, n	18(90%)	23(65.7%)	0.027*

Note: Data given as the mean ± SD; TOF, tetralogy of Fallot; PR, pulmonary regurgitation.

*P < 0.05.

### Measurements of RV and PA in patients with trans-valve surgery

The 41 patients who had received trans-valve surgery were further divided into PR and non-PR groups. The PA parameters including McGoon ratio (2.16 ± 0.50 vs. 1.67 ± 0.53; p < 0.001), Nakata index (391.29 ± 137.39 mm^2^/m^2^ vs. 268.60 ± 77.63 mm^2^/m^2^; p < 0.0001), and left pulmonary artery (LPA) diameter (20.96 ± 5.99 mm/m^2^ vs. 15.89 ± 5.96 mm/m^2^; p < 0.05) in the PR group were significantly higher compared with those in the non-PR group. No significant statistical difference was observed with respect to MPA (21.88 ± 6.96 mm/m^2^ vs. 21.63 ± 7.79 mm/m^2^) and RPA diameter (17.88 ± 5.44 mm/m^2^ vs. 16.56 ± 4.60 mm/m^2^) (Table 2).

**Table 2:** Measurements of right ventricular and pulmonary artery by DSCT compared to post-operative pulmonary regurgitation in patients with trans-valve surgery.

	PR (n = 18)	non-PR (n = 23)	Independent t test (*p*)	Spearman coefficient (*r*)
**Right Ventricle**				
RVLD, mm/m^2^	60.64 ± 15.47	72.64 ± 14.95	0.004	−0.457**
RVSD, mm/m^2^	34.89 ± 6.50	39.48 ± 7.60	0.031	−0.341*
RV wall thickness, mm/m^2^	18.61 ± 3.35	18.88 ± 3.88	0.478	−0.112
RVOT diameter, mm/m^2^	9.51 ± 3.63	10.06 ± 4.52	0.958	0.008
RVOT length, mm/m^2^	26.50 ± 10.11	23.42 ± 9.06	0.358	0.145
**Pulmonary Artery**				
MPA, mm/m^2^	21.88 ± 6.96	21.63 ± 7.79	0.834	−0.033
LPA, mm/m^2^	20.96 ± 5.99	15.89 ± 5.96	0.022	0.361**
RPA, mm/m^2^	17.88 ± 5.44	16.56 ± 4.60	0.885	0.023
McGoon ratio	2.16 ± 0.50	1.67 ± 0.53	0.009	0.413**
Nakata index, mm^2^/m^2^	391.29 ± 137.39	268.60 ± 77.63	0.002	0.482**

Note: Data given as the mean ± SD. RV, Right ventricle; RVLD, right ventricular length diameter; RVSD, right ventricular short diameter; RVOT, right ventricular outflow tract; MPA, main pulmonary artery; LPA, left pulmonary artery; RPA, right pulmonary artery.

*p < 0.05; **p < 0.01.

Regarding the RV parameters, the right ventricular length diameter (RVLD: 60.64 ± 15.47 mm/m^2^ vs. 72.64 ± 14.95 mm/m^2^; p < 0.001) and the right ventricular short diameter (RVSD: 34.89 ± 6.50 mm/m^2^ vs. 39.48 ± 7.60 mm/m^2^; p < 0.05) were significantly higher in the PR group compared with those in non-PR group. No significant difference was observed with respect to RV wall thickness (18.61 ± 3.35 mm/m^2^ vs. 18.88 ± 3.88 mm/m^2^), RVOT diameter (9.51 ± 3.63 mm/m^2^ vs. 10.06 ± 4.52 mm/m^2^), and RVOT length (26.50 ± 10.11 mm/m^2^ vs. 23.42 ± 9.06 mm/m^2^) (Table 2).

### Relationship between measurements and PR

For TOF patients who underwent tans-valve surgery, there was a positive correlation between PA parameters (LPA diameter, McGoon ratio, and Nakata index) and the occurrence of post-operative PR (r = 0.361, 0.413, and 0.482, respectively; all p < 0.05).

The diameters of the right ventricle (RVLD and RVSD) exhibited a negative correlation with PR (r = −0.457 and −0.341, respectively; all p < 0.05) (Table 2).

**Figure 1:**
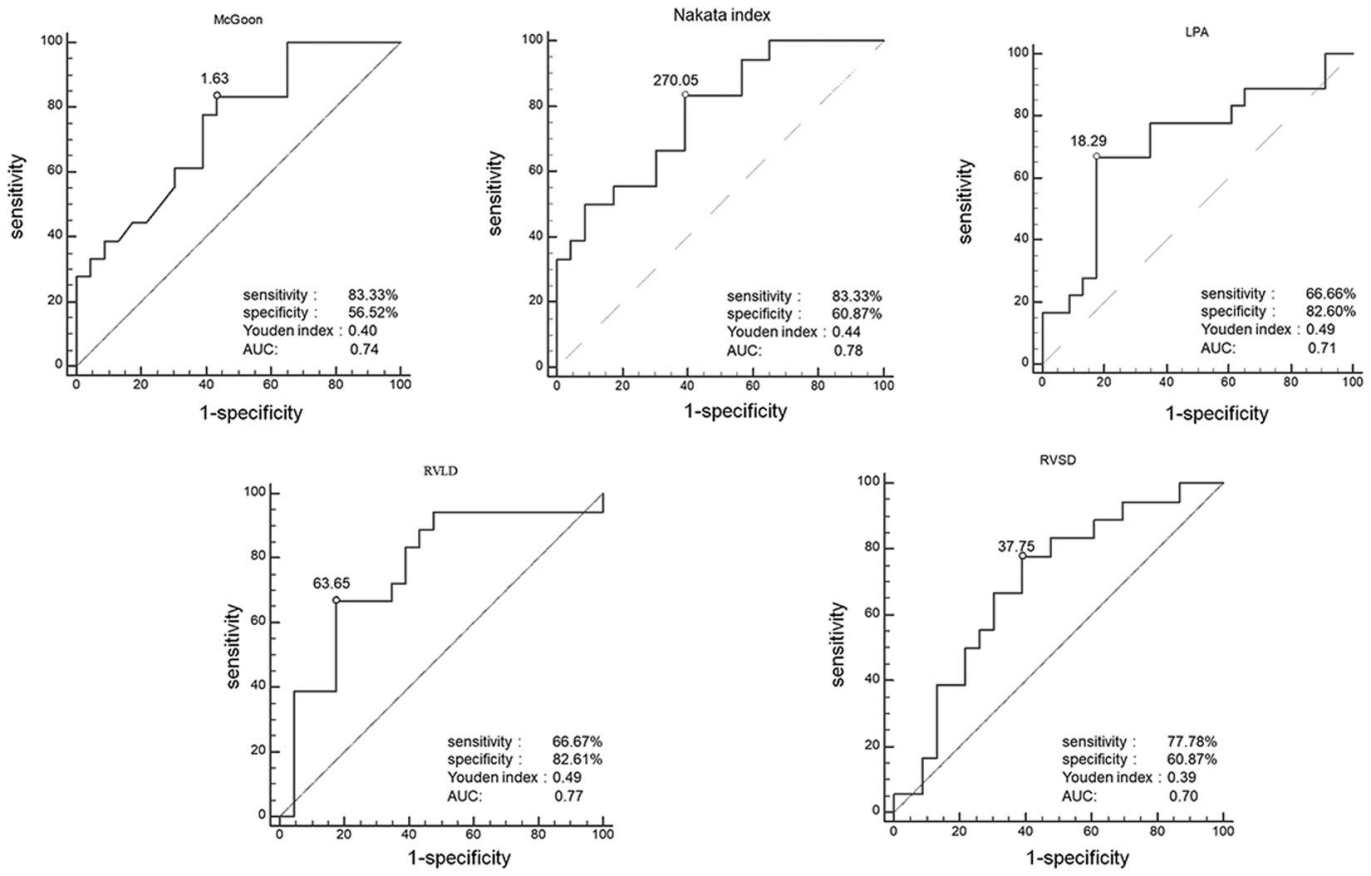
The ROC analysis to predict the relationship between the pulmonary regurgitation and measurements. In ROC analysis, the sensitivity and specificity of McGoon ratio (McGoon ratio >1.63) with DSCT for predicting pulmonary regurgitation in TOF patients were 83.3% and 56.5%, the values for Nakata index (Nakata index > 270.05) were 83.3% and 60.8% and the values for LPA diameter (LPA diameter >18.29) were 66.7% and 82.6%. The RVLD value <63.65 and RCSD value <37.75 as moderate sensitivity cutoff values (RVLD, sensitivity 66.7%, specificity 82.6%; RVSD, sensitivity 77.8%, specificity 60.9%).

ROC analysis showed that a McGoon ratio >1.63, Nakata index >270.05, and LPA diameter >18.29 based on DSCT were optimal cutoff values that predicted the risk of post-operative PR [McGoon ratio: sensitivity 83.3%, specificity 56.5%, and area under the curve (AUC): 0.74; Nakata index: sensitivity 83.3%, specificity 60.9% (AUC: 0.78); LPA: sensitivity 66.7%, specificity 82.6% (AUC: 0.71)]. The Youden index of the three parameters (McGoon ratio, Nakata index and LPA diameter) was 0.40, 0.44 and 0.49, respectively (Fig. 1).

As for RV parameters, the RVLD <63.65 and RCSD <37.75 showed a moderate sensitivity for predicting post-operative PR [RVLD: sensitivity 66.7%, specificity 82.6% (AUC:0.77); RVSD: sensitivity 77.8%, specificity 60.9% (AUC:0.70)]. The Youden index of RVLD and RVSD were 0.49 and 0.39 (Fig. 1).

### Inter-observer and intra-observer variability

Two experienced radiologists finished the intra- and inter-observer variability and all the indexes and size values using DSCT were calculated. The ICCs of intra-observer variability for the measurements were 0.878–0.997; the ICCs of inter-observer variability for the measurements were 0.824–0.996 (Table 3).

**Table 3:** Interobserver and Intraobserver variability of diameters and indexes.

	Interobserver Variability		Intraobserver Variability	
	PR	No PR	PR	No PR
RVLD	0.991	0.997	0.995	0.994
RVSD	0.878	0.929	0.824	0.983
RV wall thickness	0.978	0.978	0.966	0.978
RVOT diameter	0.989	0.992	0.988	0.995
RVOT length	0.935	0.993	0.946	0.995
McGoon ratio	0.965	0.984	0.973	0.980
Nakata index	0.966	0.983	0.998	0.982
MPA	0.993	0.997	0.995	0.996
LPA	0.997	0.995	0.995	0.994
RPA	0.985	0.983	0.993	0.992

Note: Abbreviations as in Table 2.

### Radiation dose estimation

As infant patients are susceptible to ionizing radiation, the radiation dose in our study was as low as could be reasonably achieved. The mean DLP for patients between 4 months and 1 year of old was 46.63 ± 24.81 mGy·cm; the mean ED based on DLP was 1.26 ± 0.62 mSv. The mean DLP for patients between 1 and 6 years of old was 37.29 ± 15.12 mGy·cm and the mean ED was 0.67 ± 0.27 mSv.

## Discussion

In this study, we demonstrated that PR is more likely to occur in patients who have undergone trans-valve surgery (90.0%). Pre-surgical PA measurements including the McGoon ratio, Nakata index and LPA diameter had a positive correlation with PR. Meanwhile, the right ventricle diameters (RVLD and RVSD) had a negative correlation with PR. We postulated that these PA indexes and RV diameters might serve as novel predictors of PR and facilitate clinical intervention and management for TOF patients.

TOF is the most common cyanotic CHD and accounts for 7–10% of all CHD^14^. Infant patients with TOF usually require early surgical repair to reduce the risk of death^15,16^. The surgery includes the infundibulotomy with or without trans-valve repair. PR is the most serious complication after the surgical repair, persistent PR might lead to RV dilatation, ventricular arrhythmias, and even cardiac event decades^10,17–21^. ACC/AHA 2008 Guidelines for the Management of Adults with Congenital Heart Disease point out that pulmonary valve replacement (PVR) is reasonable in adults with previous tetralogy of Fallot, severe pulmonary regurgitation, and any of the following: a. moderate to severe RV dysfunction or RV enlargement; b. development of symptomatic or sustained arrhythmias; c. moderate to severe tricuspid regurgitation^22^. The early interventions for patients who are at higher risk of post-operation PR may help to prevent or delay the occurrence of these cardiovascular events. Therefore, it is important to identify appropriate predictive parameters to improve the post-operative follow-up and clinical management of TOF patients. This study was designed to assess the influence of pre-operative RV and PA parameters on post-operative PR in infant TOF patients by DSCT.

None of the patients had PR before surgery, however, immediate post-operative TTE showed the presence and different degrees of regurgitation. Patients with trans-valve surgery presented a higher probability of PR (90.0% vs. 65.7%). Although the mechanism of development of PR is not completely understood. One of proposed cause is the ventriculotomy incision crossing the pulmonic valve annulus, a surgical process considered in patients in whom the pulmonary valve annulus is too small^23^. Our results are consistent with this theory and we assumed that the injury to the pulmonary valve and the surrounding tissue during the trans-valve incision might be the culprit.

For patients who received trans-valve surgery, the McGoon ratio, Nakata index, and the LPA diameter shown a positive correlation with PR. The above artery development parameters reflect the degree of distal stenosis of pulmonary bifurcation and a McGoon ratio higher than 1.2 and a Nakata index higher than 150 mm^2^/m^2^ are considered as essential indicators for one-stage surgery. A previous study mentioned that larger and expansile PA exhibited a tendency for higher PR fractions; the author proposed that PR was exacerbated by PA compliance but was limited by more proximal resistance^23^. Similarly, in our study, the McGoon ratio value and Nakata index value were significantly higher in the patients who developed PR. The expanded PA might attenuate the vascular elasticity and lead to reduced PA compliance. What’s more, the blood flowing through the PA is venous blood; however, the PA lacks an intravenous valve. We assumed that the well-developed and even expanded PA streams back more blood to the surgical incision at the PV level, which results in instant PR post-operatively; further, the relatively narrow valve compared with the large diameter of the pulmonary bifurcation adds to this possibility. The development parameters of PA not only described the severity of the patient’s condition, but also considered as a new predictive factor for possible PR occurring after repair surgery. However, whether the late PR is also attributable to this phenomenon is worthy of further investigation.

In our results, the LPA diameter was a better predictor of PR than RPA. The LPA diameter value was significantly different between these two groups and showed a moderate positive correlation with the presence of PR. The ROC analysis demonstrated a moderate predictive value of LPA diameter (AUC:0.71). However, the RPA diameters was not significantly different between these two group, and it did not show a significant correlation with the PR. The phenomenon might be explained as follows: a great amount of blood that streams back toward the heart is largely from the LPA^24^, and the contribution of LPA to total regurgitation flow is far greater compared with that of RPA^25^. In the event of compensatory PR after the trans-valve surgery, more blood should stream from the wider LPA back to the relatively narrow pulmonary annulus as compared with that from RPA.

Another finding of this study was that RV linear dimensions were also related to PR. The RVLD and RVSD both showed a negative correlation with the PR. Different form the size of PA, the size of RV reflects the pre-load of the pulmonary valve, namely a relatively smaller size of RV represents a lower diastolic and systolic volume before the surgery. We presume that the smaller volume may make it harder to compensate the abrupt increase in blood flow after repair and the relatively higher pressure would at least result in transient PR. However, our measurements pertained only to the early stage of post-operative period. The hemodynamic characteristics reflected in the pre-operative RV parameters and how changes in these measures on the long follow-up may provide more clues on this issue.

In our study, there was a good reproducibility of the inter- and intra-observer variabilities. With the fast scanning speed and high temporal resolution, the DSCT can provide more accurate information for PA and RV parameters. The accurate measurements can not only reflect the patients’ individual details, but also help to predict the occurrence of post-operative PR in TOF patients. PR is the most common complication after the first TOF surgical repair and is especially associated with the trans-valve surgery, however, most of patients can tolerate for several years or even decades. In the event of severe PR, the surgeon should suggest the patients to undergo the PVR^8,26^. Therefore, we may help predict the risk of PR after the one-stage operation using DSCT. The surgeon should recommend regular follow-up examination of these patients in the post-operative period.

There were several limitations in this study. First, there was a likely assembly bias. Patients selected for DSCT were all from our institution. Second, only pre-operative and immediate post-operatively changes were assessed; continued follow-up of these patients is required to determine the long-term predictive value. Third, although the radiation dose was prominently reduced through DSCT, and much lower than cardiac catheterization, infant patients are still vulnerable to ionizing radiation.

In conclusion, this study demonstrated that PA developmental parameters and RV size might be associated with post-operative PR. An increase in the LPA diameter, McGoon ratio, and Nakata index or smaller size of RV may indicate an increased risk of PR. These easily acquired pre-operative DSCT measurements can not only be used as surgical indications, but also have a certain correlation with PR incidence; these may be considered as anatomic predictive factors for the occurrence of PR.

## Materials and Methods

### Study population

This retrospective study collected 98 patients with DSCT confirmed by TOF at our hospital from January 2011 to September 2016. The exclusion criteria included (a) no-surgical patients or surgery after the age of six, (b)surgical results confirmed as not TOF, (c)incomplete clinical data, (d). Patients with poor clinical conditions. Finally, 55 patients (29 males and 26 females) with an average age of 1.67 ± 1.32 years (range: 5 months–6 years) were included in the study. Of the 55 patients, there are 41 patients received infundibulotomy with an incision across the pulmonary valve annulus, and the other 14 patients without pulmonary valve incision. This study was approved by the institutional review board of West China hospital (No. 14–163), and we pledged to abide by the declaration of Helsinki (2000 EDITION) in accordance with the relevant medical research rules of China in the study. Ensure that all patients and patients’ parents signed the relevant informed consent, and having been informed of potential adverse reactions to the iodinated contrast agent and radiation.

### Dual-source computed tomography

All examinations were performed using a DSCT scanner (Somatom Definition; Siemens Medical Solutions, Forchheim, Germany). Short-term sedation (concentration: 10%, 0.5 ml/kg) was achieved by intravenous injection of chloral hydrate prior to the cardiac DSCT examinations. Scanning was performed using a retrospective ECG-gated protocol with the following acquisition parameters: tube voltage of 80 kV, tube current of 100 mAs, gantry rotation time of 0.28 s, and pitch of 0.2–0.5 (according the heart rate with a higher pitch used for higher heart rates). The ECG-pulsing window was set on Auto. Scanning was performed in the craniocaudal direction from the inlet of the thorax to 2 cm below the diaphragm level with a scan cycle including systolic and diastolic phases. The nonionic contrast agent (370 mg/ml iopamidol; Bracco, Italy) was injected into an antecubital vein at a rate of 1.2–2.5 ml/s, followed by 20 ml of saline solution at the same flow rate. The injected volume was adjusted according body weight (1.5 ml/kg). Bolus tracking was used in the region of interest (ROI) in the descending aorta with a predefined threshold of 100 HU. Image acquisition was triggered following a delay of 5 s when the ROI attenuation threshold reached 100 HU. The workstation (Syngo; Siemens Medical System, Forchheim, Germany) processed all acquired data. A slice thickness of 0.75 mm and an increment of 0.75 mm were chosen for image reconstruction.

### Echocardiography

All patients underwent TTE using a Philips SONOS 7500 ultrasound system (Philips Medical Systems, Bothell, WA, USA) pre-operatively and immediately after surgical repaired to detect the occurrence of PR. The examination included M-mode, two-dimensional, continuous wave, and Color Doppler Flow Imaging as recommended by the American Commission on echocardiography. Post-operative PR was detected by color Doppler flow mapping and continuous wave Doppler, and the results presented as a binary carriable (present and not present). All the TTE images and detection of PR were acquired by an experienced echocardiographer.

### DSCT Image analysis

Two experienced radiologists analyzed each subject, recorded the measurements, and calculated the index. All diameters were measured using the computer caliper during systole. The multiple planar reconstruction (MPR) was used for DSCT image analysis. Recordings and assessments of RV and PA followed the American Society of Echocardiography guidelines^27^.

**Figure 2:**
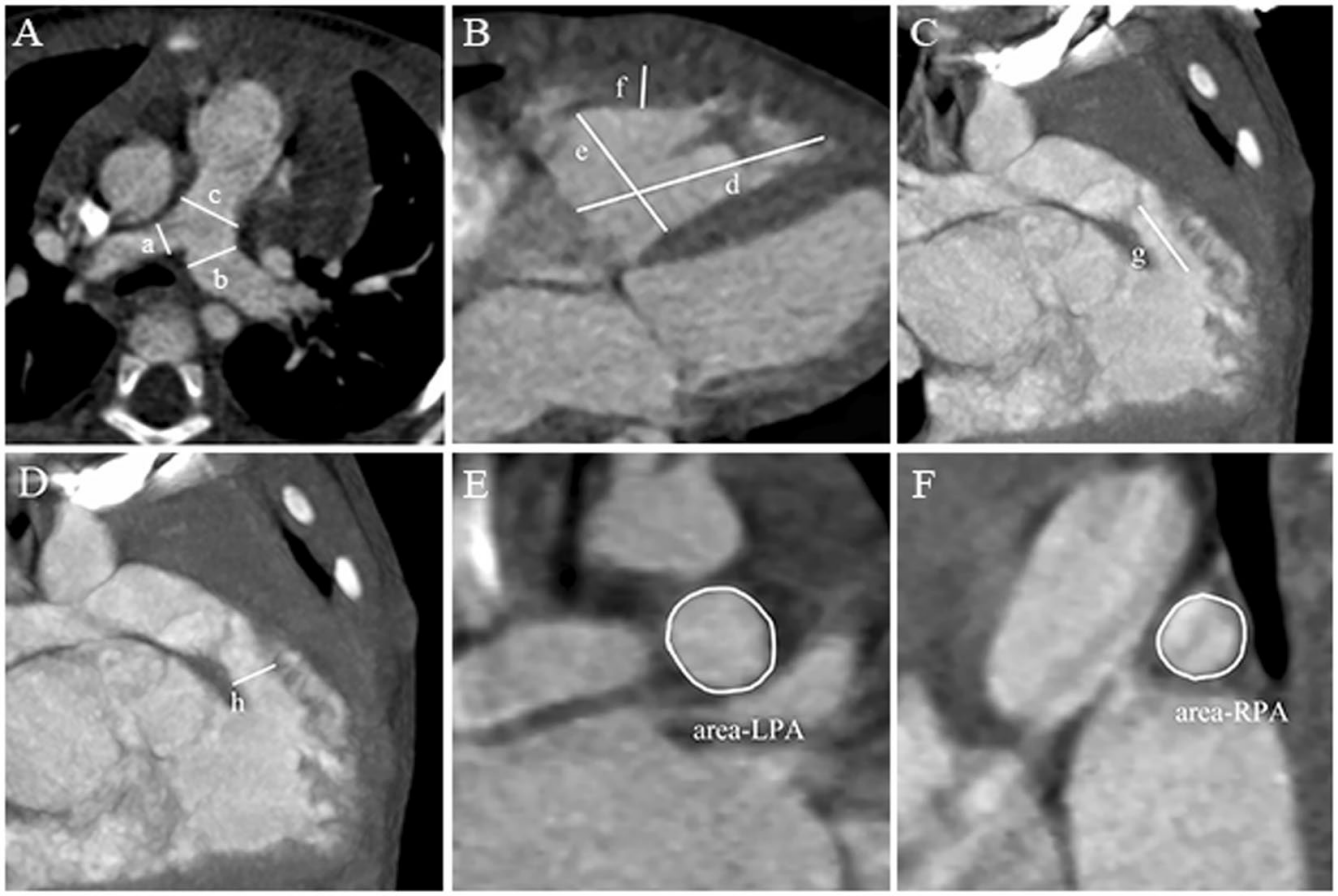
Measurements of pulmonary artery and right ventricle. Measurements of pulmonary artery (**A**): PRA diameter (D_ rpa), LPA diameter (D_ lpa), and MPA diameter (D_ mpa) were assessed on the DSCT at the level of the pulmonary artery bifurcation. The size of RV was measured from the four-chamber view (**B**). The RVOT length (L-rvot) and RVOT diameter (D_rvot) were measured from the anterior aortic wall above the aortic valve to the pulmonary valve (**C**,**D**). The cross-section areas of LPA and RPA were measured at the first branching point of each artery (**E**,**F**).

The main pulmonary artery (MPA) was measured at the site of pulmonary bifurcation in the axial slices, while the right pulmonary artery (RPA) and left pulmonary artery (LPA) were measured proximal to the first branching point of each artery (Fig. 2A). RV wall thickness was measured from the subcostal four-chamber view, preferably at the level of the tip of the anterior tricuspid leaflet. The size of RV was measured from an apical four-chamber view (Fig. 2B). The RVOT proximal dimension can be measured from the anterior aortic wall to the RV free wall above the aortic valve and immediately under the pulmonary valve respectively (Fig. 2C,D). The McGoon ratio was calculated using the equation of left and right pulmonary arterial diameter summation divided by the aorta diameter at diaphragm level. The Nakata index was calculated by the summation of LPA, RPA, and collateral vessels cross-section areas divided by body surface areas (Fig. 2E–F). The first radiologist completed all the image analyzes and measurements, then repeated the measurements 2 weeks later to determine the intra-observer variability of DSCT measurements. For the inter-observer variability, a second radiologist blindly repeated the image analyzes and measurements. The occurrence of PR was observed by the echocardiographic investigator immediately after surgery.

### Radiation dose estimation

Radiation dose parameters, including volume CT dose index (CTDIvol) and dose-length product (DLP), were automatically displayed on the CT console after the examination. Infant-specific DLP conversion coefficients, based on the 2007 recommendations of the International Commission on Radiological Protection, were used to calculate the effective dose (ED) using conversion coefficients of 0.039 for patients <4 months of age, 0.026 for patients between 4 months and 1 year of age, and 0.018 for patients between 1 and 6 years of age.

### Statistical analysis

The data were analyzed using the SPSS software for Windows (version 19.0, SPSS Inc., Chicago, IL, USA) and MedCalc software (version 9.3.0.0, MedCalc software, Mariakerke, Belgium). The McGoon ratio and Nakata index were calculated using PA diameters and cross-section areas. Continuous variables were expressed as mean ± standard deviations (SD). Categorical variables were expressed as numbers. The significant differences of these measurements between the PR and non-PR group in trans-valve surgical group were analyzed using an non-parametric test. Spearman’s rank correlation test was used to evaluate the association between these pre-operative parameters and the occurrence of PR. Receiver operating characteristic (ROC) analyses calculated the sensitivity and specificity of the effective parameters to predict post-operative PR. The intraclass correlation coefficient (ICC) was used to assess the inter-observer and intra-observer variability. A two-tailed p-value of <0.05 was considered statistically significance.
